# Ventricular cardiac magnetic resonance traits and schizophrenia risk: a UK Biobank and Mendelian randomization study

**DOI:** 10.3389/fpsyt.2026.1766780

**Published:** 2026-06-12

**Authors:** Jinfeng Yan, Siqi Yu, Yuan Gao, Jiajing Cai, Zhenghao Deng, Jian Yu, Qidong Liu

**Affiliations:** 1 Shanghai Engineering Research Center of Tooth Restoration and Regeneration & Tongji Research Institute of Stomatology & Shanghai Tongji Stomatological Hospital and Dental School, Tongji University, Shanghai, China; 2 Bacteriological laboratory, Linyi Center for Disease Control and Prevention, Linyi, Shandong, China; 3 Independent Researcher, Boston, MA, United States; 4 Center of Advanced Medical Computing and Analysis, Massachusetts General Hospital and Harvard Medical School, Boston, MA, United States; 5 School of Medicine, Tongji University, Shanghai, China

**Keywords:** genome-wide association study, Mendelian randomization, polygenic risk scores, schizophrenia, ventricular CMR traits

## Abstract

**Background:**

Genetic factors can influence variation in cardiac structure. Schizophrenia (SCZ) is also highly heritable. However, whether cardiac structural variation is related to SCZ risk remains unclear and insufficiently examined from a "heart-to-brain" perspective.

**Methods:**

Using data from 38,206 UK Biobank (UKB) participants, we assessed associations between 16 cardiac magnetic resonance imaging-derived ventricular traits and SCZ polygenic risk scores. Using ventricular cardiac magnetic resonance traits Genome-wide association study (GWAS) data from over 30,000 individuals and SCZ GWAS data from 127,906 individuals, two-sample Mendelian randomization (MR) was performed to evaluate potential causal associations. Reverse association analysis using data from UKB and reverse MR analysis were conducted to explore possible reverse effects.

**Results:**

Four ventricular traits showed significant associations with SCZ genetics, with end-systolic volume (ESV) of right ventricle (RV) exhibiting the strongest negative association. Forward MR provided evidence consistent with a potential causal association between genetically predicted higher ESV of RV and lower SCZ risk, with no strong indication of pleiotropy. All other traits in the forward MR analyses, as well as all traits in the reverse MR analyses, showed no statistically significant evidence of a potential causal association.

**Conclusions:**

Higher genetically predicted ESV of RV appears linked to reduced SCZ risk. These findings highlight ESV of RV as a ventricular characteristic with genetic relevance to SCZ. This study offers new insights into SCZ pathophysiology and opportunities for risk stratification and mechanistic investigation.

## Background

In recent years, brain-heart interaction has emerged as a research focus in neuroscience and cardiovascular studies (1–5). Growing evidence suggests that alterations in cardiac structure are not merely consequences of cardiovascular disease, but may also be associated with changes in brain function and mental health through potential “heart-to-brain” interactions (6–8). For example, heart failure is linked to cognitive impairment and neurodegenerative changes (7). Genetic studies have identified shared genetic components and significant genetic correlations between cardiac structural traits and neuropsychiatric disorders (9–13). Ventricular structural alterations are key features of cardiac disorders and are significantly influenced by genetic factors (14–17). Among psychiatric disorders, schizophrenia (SCZ) is a highly heritable psychiatric disorder (18–20). Notably, studies have shown that individuals with SCZ face a higher risk of cardiovascular disease compared to the general population, suggesting a potential link between cardiac structural alterations and SCZ (21–25). However, several important limitations remain. First, most existing studies have focused on the comorbidity between cardiovascular disease and SCZ rather than potential causal associations (23–25). Second, current evidence primarily emphasizes the impact of SCZ on cardiac function, whereas the potential influence of cardiac structural alterations on SCZ risk remains poorly understood (24, 25). Finally, whether shared genetic factors contribute to both cardiac phenotypes and SCZ has not been fully elucidated (24). Therefore, there is an urgent need to further investigate the potential influence of ventricular structural alterations on the risk of developing SCZ, which may provide insights into disease mechanisms and therapeutic strategies.

In this study, we first analyzed data from the UK Biobank (UKB) to systematically examine the relationship between ventricular cardiac magnetic resonance (CMR) traits and SCZ polygenic risk scores (PRS), which showed evidence that approximately half of the ventricular CMR traits were associated with SCZ PRS. To further investigate the potential causal relationship between ventricular CMR traits and SCZ risk, we performed Mendelian randomization (MR) analysis, a genetic epidemiological approach that uses germline variants as instrumental variables to infer potential causal associations between exposures and outcomes (26, 27). Forward MR analysis showed that genetically predicted increases in end-systolic volume (ESV) of right ventricle (RV) were associated with a reduced risk of SCZ. This association remained robust across multiple sensitivity analyses. Additionally, reverse MR and supplementary UKB analyses indicated an association between SCZ and ESV of RV. However, the reverse MR result was not statistically significant after multiple-testing correction. Therefore, the overall evidence provides stronger support for the “ventricular structure–to–SCZ” direction. Overall, this study provides novel evidence at the genetic and causal inference levels, suggesting that ventricular structural features, particularly ESV of RV, may influence the risk of SCZ through a heart-to-brain pathway. These findings broaden the current understanding of psychiatric disorders and suggest that cardiac structural traits may potentially contribute to SCZ susceptibility, offering potential implications for future therapeutic strategies.

## Methods

### Ethics declarations

All data used in this study were obtained from the UKB and publicly available genome-wide association study (GWAS) summary statistics; thus, no additional ethical approval or informed consent was required.

### UKB analysis

#### Recruitment and consent in UKB

Participants included in this observational study were drawn from the UKB, an ongoing population-based cohort comprising approximately 500,000 community-dwelling individuals aged 40–69 years, who were recruited across Great Britain between 2006 and 2010. Recruitment was conducted through invitations sent via the National Health Service (NHS) registry, and individuals voluntarily enrolled in the study. Beginning in 2014, a subset of participants was further invited to participate in the imaging sub-study based on random selection procedures implemented by the UKB (28). All participants were screened according to predefined criteria and provided written informed consent prior to participation in accordance with UKB protocols. The UK Biobank project received ethical approval from the National Research Ethics Service North West (11/NW/0382), and all participants provided written informed consent for participation and data use within the UKB framework. Detailed information regarding recruitment procedures, assessment protocols, and consent processes is publicly available through the UKB website (http://www.ukbiobank.ac.uk).

#### Inclusion and exclusion criteria for UKB participants

Specifically, participants were eligible for inclusion if they met the following criteria: (1) availability of complete data for all eight ventricular CMR traits derived from UKB imaging assessments; (2) availability of complete SCZ PRS data from UKB; and (3) availability of complete information for all predefined covariates included in the regression models, including age, sex, ethnicity, education, assessment center, socioeconomic status, smoking status, and alcohol consumption. Participants with missing data for any of the above variables were excluded from the final analysis to ensure consistency and completeness of the statistical analyses.

### Databases of ventricular CMR traits and SCZ PRS

For ventricular CMR traits, we used the data from UKB Category 102, Field-ID 24100–24103 and Field-ID 24106-24109, including end-diastolic volume (EDV), ESV, stroke volume (SV), and ejection fraction (EF) of left ventricle (LV) or RV. For SCZ PRS, we used the standard PRS data provided by UKB Category 301, Field-ID 26275. The standard PRS was calculated on all individuals in UKB and is built from external GWAS training data only. Details on ventricular CMR traits and SCZ PRS are provided in **Supplementary Table 1**. The sample size of the present study was comparable to those of previously published studies on similar topics, supporting the adequacy of the study population for the present analyses (29, 30).

### Acquisition and processing of ventricular CMR traits data

Ventricular CMR imaging data were obtained from the UKB imaging dataset using a standardized acquisition protocol (31, 32). All CMR examinations were performed on a 1.5-T scanner (MAGNETOM Aera, Siemens Healthcare, Erlangen, Germany) with electrocardiographic gating and balanced steady-state free precession cine sequences, according to the established UKB imaging protocol. The imaging protocol included long-axis and short-axis cine acquisitions covering both the left and right ventricles. Ventricular functional parameters were automatically derived from the CMR images using the UKB imaging processing pipeline.

### Acquisition and processing of ventricular SCZ PRS data

SCZ PRS was generated by UKB using summary statistics from a large external genome-wide association study (GWAS) meta-analysis of SCZ, including 97,456 cases and 334,331 controls, while UKB participants were used as the target dataset (33, 34). A Bayesian modeling framework integrating information across multiple ancestries and genetically correlated traits was applied to estimate posterior effect sizes for genetic variants across the genome. Individual SCZ PRS values were subsequently calculated as the weighted sum of SCZ-associated alleles carried by each participant. To ensure data quality and genetic reliability, variants included in PRS construction were required to meet multiple quality-control criteria, including high imputation quality, consistency of allele frequency across reference populations, conformity with Hardy-Weinberg equilibrium, and successful mapping between genome builds. In addition, low-frequency variants, indels, and variants located within pseudoautosomal regions were excluded. The resulting raw PRS values were further standardized by UKB to account for ancestry distribution differences across participants.

### Covariates

Baseline covariates were selected based on their potential role as confounders in the association between SCZ and ventricular CMR traits, as they are well-recognized factors that may influence both cardiac traits and the risk of SCZ. Such covariates encompass demographic variables, such as age at imaging; sex (female or male, Field-ID 31); ethnicity (white or nonwhite, Field-ID 21000); education (college, other levels, or unknown; Field-ID 6138); assessment center (Field-ID 54); socioeconomic status (Field-ID 22189, Townsend deprivation index) (24, 35, 36). Health associated covariates comprised smoking status (never, previous, or current; Field-ID 20116) and alcohol consumption (never, previous, or current; Field-ID 1558) (37–40).

### Statistics

Multivariate linear regression models were constructed to evaluate the association between ventricular CMR traits and SCZ PRS. All models were adjusted for the aforementioned baseline covariates, including age at imaging, sex, ethnicity, education, assessment centre, socioeconomic status, smoking status, and alcohol consumption.

### MR analysis

#### Databases of ventricular CMR traits and SCZ

For ventricular CMR traits, GWAS statistics were obtained from Schmidt et al. (2023), a large-scale MR study measured in up to 36,548 UKB subjects (41). Sample size for each ventricular CMR trait exceeds 30,000, and detailed sample sizes for ventricular CMR traits are provided in **Supplementary Table 2**. For SCZ, GWAS statistics were obtained from Trubetskoy et al. (2023), large-scale GWAS statistics of SCZ including 175,799 individuals (42). We selected SCZ GWAS summary statistics derived from individuals of European ancestry, with a total sample size of 127,906, including 52,017 cases and 75,889 controls. Detailed information regarding these GWAS datasets is provided in **Supplementary Table 3**.

#### MR analysis design

In order to evaluate the influence of ventricular CMR traits on SCZ outcome, we took the forward MR analysis, using the GWAS of ventricular CMR traits that regressed ventricular CMR traits on genotype, as the exposure dataset. To evaluate the potential reverse causal relationship, we took the reverse MR analysis, using ventricular CMR traits as the outcome and employed the GWAS of SCZ, where SCZ was regressed on genotype as the exposure dataset. All data used were publicly available GWAS summary statistics and no individual-level data were analyzed.

#### Selection of IVs

We initially identified lead Single Nucleotide Polymorphisms (SNPs) linked to ventricular CMR traits or SCZ that reached genome-wide significance (*P* < 5 × 10^-8^) (43–45), and designated these SNPs as candidate IVs for MR analysis. To ensure the independence of IVs, linkage disequilibrium (LD) pruning was performed using a stringent threshold (r²< 0.001 within a 10,000 kb window) based on the European reference panel from the 1000 Genomes Project. Instrument strength was subsequently evaluated using the F-statistic, with IVs exhibiting F-statistics below 10 considered potentially susceptible to weak instrument bias (46, 47). The parameters required to test the *F-*statistics were *R^2^* (explained variance of genetic instruments on exposure), n (the sample size of GWAS for exposure) and k (the number of instruments). The specific formula for calculating *F*-statistics is shown below (46).


$$F=\frac{R^{2}\times \left(n-k-1\right)}{\left(1-R^{2}\right)\times k}$$

Steiger filtering was also applied to each IV-outcome pair to confirm causal direction, with only IVs showing stronger association with exposure than outcome retained (48). The final list of IVs, along with the corresponding *R^2^* values and calculated *F*-statistics for the MR analyses are provided in **Supplementary Table 4** (forward MR analysis) and **Supplementary Table 5** (reverse MR analysis).

### Confounder removal and pleiotropy control

To further minimize potential bias due to horizontal pleiotropy and confounding, all candidate IVs were systematically screened for suggestive associations (*P* < 1 × 10^-5^) with traits that could influence both exposure and outcome. In the forward MR analysis, IVs associated with smoking status, alcohol consumption, and brain-related phenotypes were evaluated, whereas in the reverse MR analysis, IVs associated with smoking status, alcohol consumption, and cardiovascular-related phenotypes were assessed. Screening was performed using the NHGRI-EBI GWAS Catalog (https://www.ebi.ac.uk/gwas/) (49, 50). SNPs showing potential associations with these confounding traits were excluded to reduce the likelihood that the observed associations were driven by alternative biological pathways independent of the exposure of interest. In addition, because immune and inflammatory pathways have been implicated in both cardiovascular traits and SCZ, IVs linked to immune- or inflammation-related biological processes were further identified and removed. Functional annotation was performed using the NHGRI-EBI GWAS Catalog and Ensembl Variant Effect Predictor (VEP) (https://grch37.ensembl.org/info/docs/tools/vep/), combined with pathway enrichment analysis using g:Profiler (https://biit.cs.ut.ee/gprofiler/). All excluded IVs and the corresponding exclusion criteria are listed in **Supplementary Table 6** (forward MR analysis) and **Supplementary Table 7** (reverse MR analysis).

### MR analysis

MR analyses were conducted to evaluate the influence of ventricular CMR traits on SCZ and subsequently examined the reverse effect. The primary analysis used the inverse-variance weighted (IVW) method, applying either a fixed-effects model or a multiplicative random-effects model depending on the degree of heterogeneity among instruments. Because the IVW approach may yield biased estimates in the presence of horizontal pleiotropy, three complementary MR methods were additionally applied to enhance the robustness and reliability of the causal inference. Specifically, the MR-Egger method estimates the causal effect using the slope coefficient of the Egger regression, which provides a more robust estimate even if none of the IVs are invalid (51). The weighted median method offers consistent estimates when up to 50% of the total instrument weight comes from valid variants (52). The weighted mode method groups instruments by similarity and estimates the potential causal association based on the largest consistent cluster (53).

### Sensitivity analysis

The robustness of statistically significant MR results was validated via multiple sensitivity analyses. First, heterogeneity across IVs was measured using Cochran’s Q statistic, where *P* > 0.05 indicated low inter-SNP heterogeneity (54). Second, horizontal pleiotropy was assessed via the MR-Egger intercept, a non-significant intercept (*P* > 0.05) implied no directional pleiotropy, meaning IVs affected the outcome mainly through the exposure (55). Finally, leave-one-out analyses were conducted by sequentially removing each SNP and recalculating the IVW effect (56). Consistent results across iterations confirmed that the overall association was not driven by any single IV. To further assess the robustness of the findings, a series of additional sensitivity analyses was performed. Firstly, to increase the number of SNPs used as IVs for ventricular CMR traits, the significance threshold for IV selection was relaxed from genome-wide significance to *P <* 1 × 10^–7^ and 1 × 10^-6^, and the forward MR analysis was conducted. Then, to account for correlated psychiatric liabilities and minimize potential horizontal pleiotropy, multivariable Mendelian randomization (MVMR) analyses were conducted with ESV of RV as the primary exposure and SCZ risk as the outcome, while GWAS summary statistics of major depressive disorder (MDD) (57), substance use disorder (SUD) (58), and post-traumatic stress disorder (PTSD) (59) were jointly modeled as covariant. The primary MVMR estimates were derived using the random-effects multivariable IVW method. Given potential heterogeneity, multivariable MR-Egger and the multivariable weighted median estimator were additionally applied.

### Data analysis of UKB and MR analyses

All analyses were performed using R Software (4.4.2). MR analyses were performed using the MendelR (60). For continuous outcomes, results were reported as β values with corresponding 95% confidence intervals (CI), whereas categorical outcomes were reported as odds ratios (OR) with 95% CI. All statistical tests were two-sided. Statistical significance was defined as *P* < 3.125 × 10^–3^ after Bonferroni correction (16 tests), while *P* < 0.05 was considered nominally significant. Causal associations were considered reliable when the IVW estimate reached statistical significance, at least two additional MR methods showed nominal significance, and effect directions were consistent across methods.

## Results

### Baseline characteristics of UKB analysis participants

The baseline characteristics of the participants are presented in **Table 1**. Among the 38,206 included participants, 52.0% were female and 48.0% were male, with a mean imaging age of 63.6 years. Most participants were White (96.9%). Regarding smoking status, 60.5% had never smoked, 33.1% were previous smokers, and 6.2% were current smokers. In terms of alcohol consumption, 95.2% were current alcohol users. Regarding education level, 46.0% had a college education and 46.8% had other educational levels. Participants were mainly recruited from the Cheadle assessment center (63.1%), followed by Newcastle (25.6%) and Reading (11.3%). The mean Townsend deprivation index, used to reflect socioeconomic status, was -1.91.

**Table 1:** Baseline characteristics of UKB analysis participants.

Characteristics		Overall (N=38206)
Sex	Female	19858 (52.0%)
	Male	18348 (48.0%)
Age at imaging	Mean (SD)	63.6 (7.56)
Ethnicity	White	37033 (96.9%)
	Nonwhite	1075 (2.8%)
	Missing	98 (0.3%)
Smoking	Current	2384 (6.2%)
	Never	23107 (60.5%)
	Previous	12633 (33.1%)
	Missing	82 (0.2%)
Alcohol	Current	36376 (95.2%)
	Never	970 (2.5%)
	Previous	841 (2.2%)
	Missing	19 (< 0.1%)
Education	College	17581 (46.0%)
	Other levels	17882 (46.8%)
	Missing	2743 (7.2%)
Assessment centre	Cheadle	24108 (63.1%)
	Newcastle	9762 (25.6%)
	Reading	4336 (11.3%)
Townsend deprivation index	Mean (SD)	-1.91 (2.71)
	Missing	37 (0.1%)

UKB, UK Biobank.

### UKB analysis identified significant associations between multiple ventricular CMR traits and SCZ PRS

**Figure 1:**
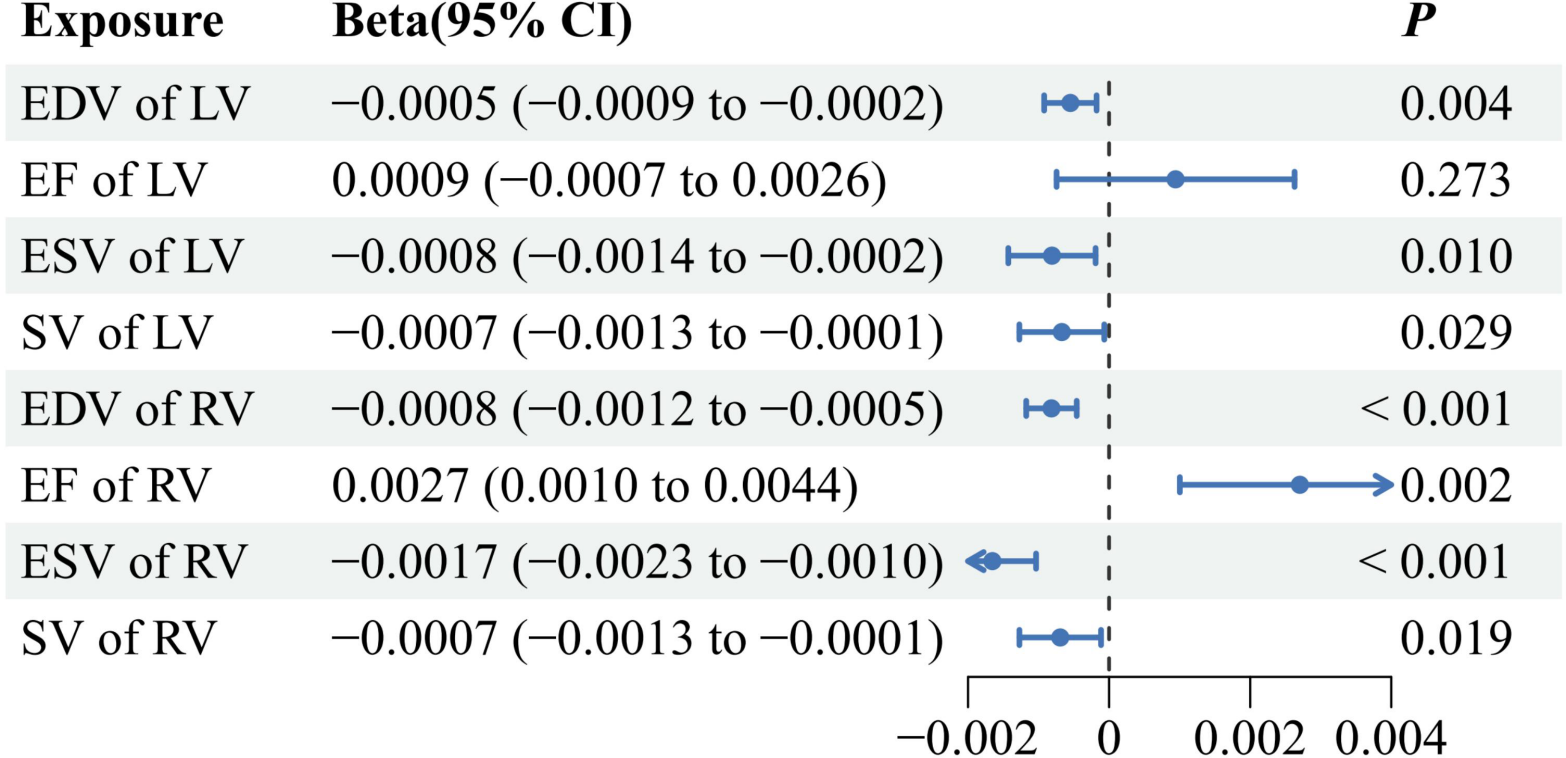
Association between ventricular CMR traits and SCZ PRS. CMR, cardiac magnetic resonance; SCZ, schizophrenia; RPS, polygenic risk scores; CI, confidence interval; EDV, end diastolic volume; EF, ejection fraction; ESV, end systolic volume; SV, stroke volume; LV, left ventricle; RV, right ventricle.

As shown in **Figure 1**, the UKB analysis revealed a significant association between four ventricular CMR traits including EDV of RV (β = -0.0008, 95% CI: -0.0012 ~ -0.0005; *P* < 0.001), EF of RV (β = 0.0027, 95% CI: 0.0010 ~ 0.0044; *P* = 0.002), and ESV of RV (β = -0.0017, 95% CI: -0.0023 ~ -0.0010; *P* < 0.001) and SCZ PRS. Among all of these ventricular CMR traits, the higher EDV of RV and ESV of RV were associated with lower SCZ PRS. In contrast, the higher EF of RV was associated with higher SCZ PRS.

### Forward MR analysis identified a potential causal association between higher ESV of RV and reduced SCZ risk

**Figure 2:**
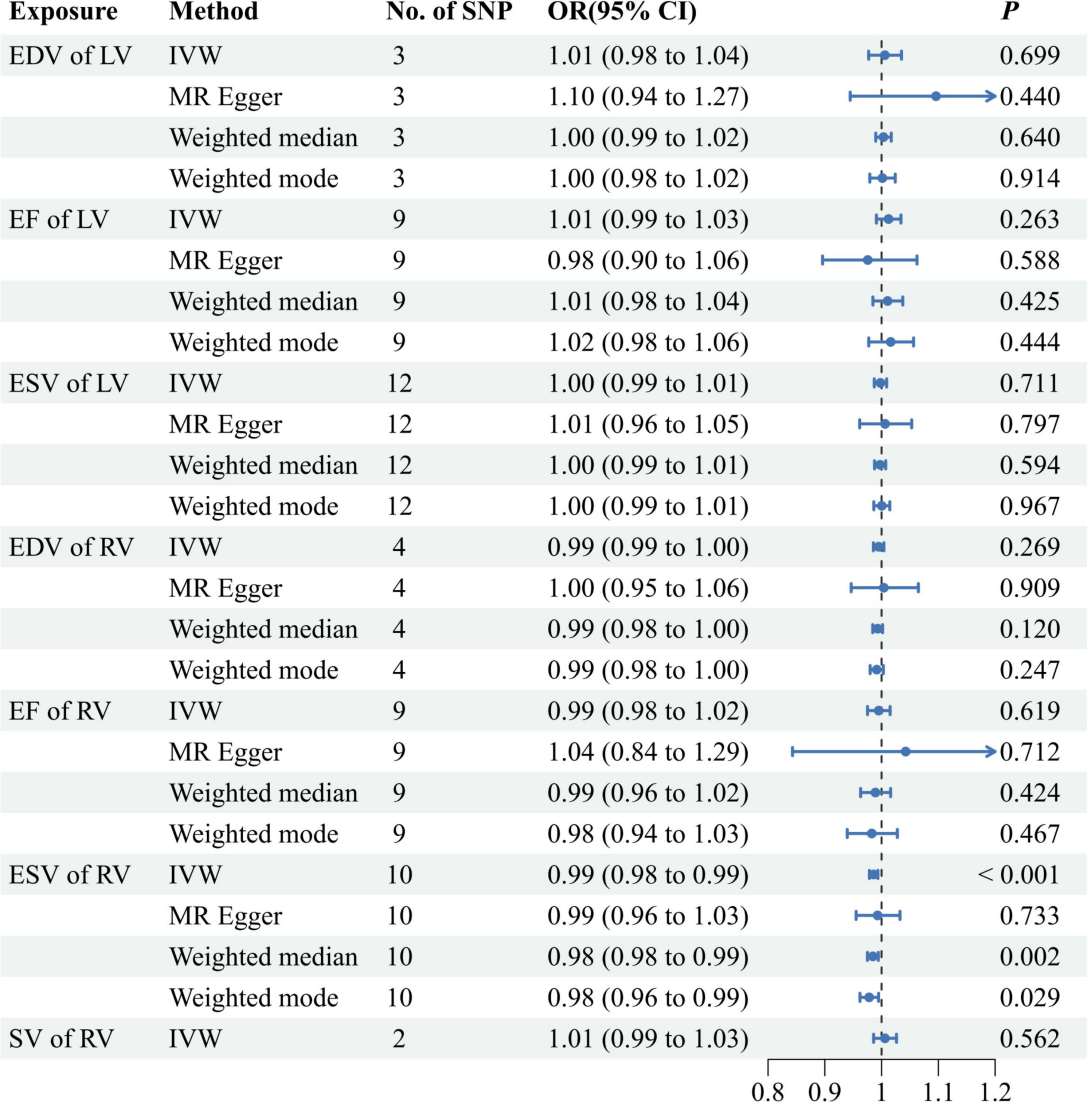
Forward MR analysis of the causal association between ventricular CMR traits and SCZ. SCZ, schizophrenia; SNP, single nucleotide polymorphism; OR, odds ratio; CI, confidence interval; EDV, end diastolic volume; EF, ejection fraction; ESV, end systolic volume; SV, stroke volume; LV, left ventricle; RV, right ventricle; IVW, inverse-variance weighted; MR, Mendelian randomization.

As shown in **Figure 2**, the forward MR analyses found that for SCZ outcome, the IVW analysis showed that ESV of RV was associated with SCZ risk (OR = 0.99, 95% CI: 0.98 ~ 0.99; *P* < 0.001). Consistent results were observed across the weighted median and weighted mode methods, both of which also reached nominal significance (*P* < 0.05). In contrast, for other ventricular CMR traits, there was no such statistical evidence (all *P* > 3.125 × 10^-3^). Consistent results were observed across the weighted median and weighted mode methods, further supporting the robustness of the findings. Thus, the higher genetically predicted ESV of RV showed evidence consistent with a potential causal association with a reduced risk of SCZ. In contrast, no significant causal associations were identified between the remaining ventricular CMR traits and SCZ risk.

### Sensitivity analyses supported the robustness of the association between ESV of RV and SCZ risk

As shown in **Table 2**, heterogeneity across instruments was not statistically significant. For the SCZ outcome, Cochran’s Q test yielded an IVW *P* of 0.303 and an MR-Egger *P* of 0.235. Also, no evidence of directional pleiotropy was detected. For the SCZ outcome, the MR-Egger intercept yielded a *P* of 0.725. Leave-one-out analysis showed that excluding individual SNPs had minimal impact on the IVW estimate, which was shown in **Supplementary Figure 1**.

**Table 2:** Heterogeneity and pleiotropy tests for the causal association between ESV of RV and SCZ.

Heterogeneity test		Pleiotropy test
Cochran’s Q Test IVW *P* Value	Cochran’s Q Test MR Egger *P* Value	MR Egger intercept *P* Value
0.303	0.235	0.725

ESV, end systolic volume; RV, right ventricle; SCZ, schizophrenia; IVW, inverse-variance weighted; MR, Mendelian randomization.

As the number of IVs for ventricular CMR traits was limited at the conventional genome-wide significance threshold (*P<* 5 × 10^-8^), the threshold was further relaxed to *P* < 1 × 10^–7^ and *P* < 1 × 10^–6^ to assess the stability of the findings. At the *P* < 1 × 10^–7^ threshold, the association between ESV of RV and SCZ remained significant (OR = 0.99, 95% CI: 0.98 ~ 1.00; *P* = 0.002), and consistent results were observed across the weighted median and weighted mode methods, both of which also reached nominal significance (*P* < 0.05) (**Supplementary Figure 2**). At the *P* < 1 × 10^–6^ threshold, both the weighted median (OR = 0.99, 95% CI: 0.98 ~ 1.00; *P* = 0.013) and weighted mode (OR = 0.98, 95% CI: 0.97 ~ 1.00; *P* = 0.039) estimates remained directionally consistent with the primary analysis, whereas the IVW estimate was not statistically significant (OR = 0.99, 95% CI: 0.98 ~ 1.00; *P* = 0.105) (**Supplementary Figure 3**). In addition, given that many IVs associated with SCZ may also be correlated with other psychiatric disorders, MVMR was performed to account for potential psychiatric comorbidities by jointly modeling MDD, SUD, and PTSD as covariates. Although the IVW and MR-Egger estimates for the association between ESV of RV and SCZ were not statistically significant in the MVMR framework (*P* = 0.454 and *P* = 0.355, respectively; **Supplementary Table 8**), heterogeneity tests indicated significant heterogeneity in both models (**Supplementary Table 8**). Therefore, the weighted median estimator was additionally applied as a robustness analysis. Under the weighted median approach, the estimated effect of ESV of RV on SCZ risk was directionally consistent with the primary analysis (OR = 0.99, 95% CI: 0.98 ~ 1.00; *P* = 0.041). Overall, these findings suggest that the association between ESV of RV and SCZ remains directionally consistent after accounting for psychiatric comorbidity traits; however, the results should be interpreted with caution in light of the substantial heterogeneity and potential pleiotropy.

Together, these findings support the robustness of the potential causal association of ESV of RV on SCZ and indicate that the result is unlikely to be driven by pleiotropy, heterogeneity, or single-instrument bias.

### Reverse MR analysis provided limited evidence for a causal effect of SCZ on ventricular CMR traits

**Figure 3:**
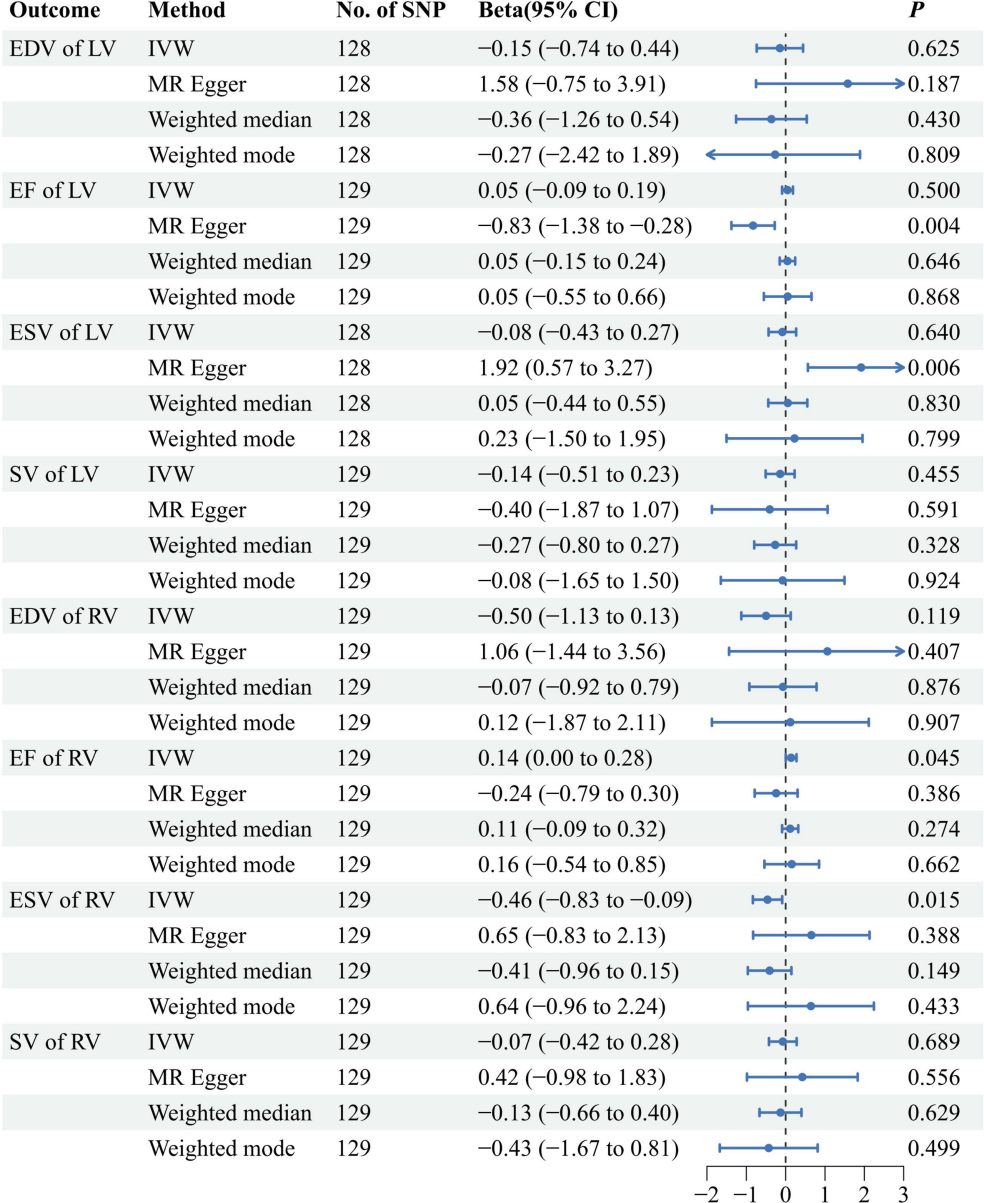
Results of the reverse MR analysis. SNP, single nucleotide polymorphism; OR, odds ratio; CI, confidence interval; EDV, end diastolic volume; EF, ejection fraction; ESV, end systolic volume; SV, stroke volume; LV, left ventricle; RV, right ventricle; IVW, inverse-variance weighted; MR, Mendelian randomization.

As shown in **Table 3**, the UKB analysis confirmed the association between SCZ PRS and ventricular CMR traits including EDV of RV, EF of RV, and ESV of RV, which was consistent with the conclusion drawn from **Figure 1**, and the tertile-stratified analysis confirmed this conclusion. As seen in **Figure 3**, the reverse MR analysis found no statistical evidence supporting a causal effect of SCZ on ventricular CMR traits (all *P* > 3.125 × 10^-3^). An association between genetically predicted SCZ and ESV of RV was observed (β = -0.46, 95% CI: -0.83 ~ 0.09; *P* = 0.015), although this finding did not survive Bonferroni correction and should therefore be interpreted with caution.

**Table 3:** Results of the reverse association analysis using data from UKB.

Outcome	SCZ PRS	*P*	SCZ PRS T1	SCZ PRS T2	*P*	SCZ PRS T3	*P*
EDV of LV	-0.40 (-0.67 to -0.13)	0.004	Reference	-0.14 (-0.80 to 0.51)	0.664	-0.88 (-1.55 to -0.22)	0.009
EF of LV	0.03 (-0.03 to 0.09)	0.273	Reference	0.01 (-0.14 to 0.15)	0.927	0.07 (-0.07 to 0.22)	0.335
ESV of LV	-0.21 (-0.38 to -0.05)	0.010	Reference	-0.07 (-0.47 to 0.32)	0.719	-0.49 (-0.89 to -0.09)	0.016
SV of LV	-0.19 (-0.35 to -0.02)	0.029	Reference	-0.07 (-0.48 to 0.33)	0.725	-0.39 (-0.80 to 0.02)	0.059
EDV of RV	-0.64 (-0.92 to -0.36)	< 0.001	Reference	-0.45 (-1.13 to 0.23)	0.195	-1.36 (-2.05 to -0.67)	< 0.001
EF of RV	0.09 (0.03 to 0.15)	0.002	Reference	0.10 (-0.04 to 0.25)	0.155	0.20 (0.06 to 0.35)	0.006
ESV of RV	-0.43 (-0.60 to -0.27)	< 0.001	Reference	-0.34 (-0.73 to 0.05)	0.088	-0.92 (-1.32 to -0.53)	< 0.001
SV of RV	-0.21 (-0.38 to -0.03)	0.019	Reference	-0.11 (-0.53 to 0.31)	0.615	-0.44 (-0.86 to -0.01)	0.044

UKB, UK Biobank; SCZ, schizophrenia; RPS, polygenic risk scores; EDV, end diastolic volume; EF, ejection fraction; ESV, end systolic volume; SV, stroke volume; LV, left ventricle; RV, right ventricle.

## Discussion

In the present study, we first conducted a UKB analysis, in which we identified statistically significant associations between nearly half of the ventricular CMR traits and SCZ PRS. Building on this, we subsequently performed forward MR analysis and found evidence that genetically predicted higher ESV of RV was associated with a reduced risk of SCZ. The robustness of this finding was supported by multiple sensitivity analyses. Additionally, to further explore the reverse relationship between ventricular CMR traits and SCZ, we performed reverse association analysis using UKB data and reverse MR analysis. These analyses suggested an association between SCZ and ESV of RV, although the reverse MR result was not statistically significant after multiple-testing correction. Overall, through a series of analyses, we found that genetically predicted higher ESV of RV was associated with a reduced risk of SCZ.

Our results provide genetic evidence consistent with the underexplored “heart-to-brain” axis, suggesting that genetic determinants of cardiac structure may have downstream consequences for neuropsychiatric disease risk. The specificity of the association with ESV of RV, rather than other ventricular parameters, may reflect distinct underlying biological processes rather than a generalized cardiac effect. Notably, high ESV of RV is typically associated with ventricular dilation and impaired systolic function, and is often associated with adverse cardiovascular outcomes (61, 62). The inverse association between genetically predicted ESV of RV and SCZ risk warrants further investigation. This finding may indicate that the relationship between cardiac structure and SCZ is not solely explained by overt cardiovascular dysfunction but could involve more complex physiological or genetic interactions linking the heart and the brain.

From a hemodynamic perspective, modest increases in ESV of RV may not be assumed to represent overt ventricular dysfunction (63), but could reflect broader physiological or compensatory cardiovascular variation (64–66). Given the close interaction between cardiovascular and cerebral systems, it is possible that RV structural variation may be related to systemic hemodynamic or cardiopulmonary processes potentially relevant to brain function (66–68). Based on the present findings, it is possible that a slightly enlarged right ventricle may increase volume reserve and buffer venous return fluctuations, and this potential stabilization in cardiac output may be associated with systemic hemodynamic variation potentially relevant to brain physiology (68, 69). Sustained cerebral blood flow and oxygen supply may influence oxidative stress, which has been implicated as a pathological factor in SCZ (70–72). Collectively, these observations raise the possibility that hemodynamic variation may be involved in SCZ-related pathophysiology. Overall, these hemodynamic considerations may partially explain the observed association between higher ESV of RV and lower SCZ risk, potentially through mechanisms related to circulation stability and cerebral perfusion.

The more prominent association of RV, rather than LV, traits with SCZ risk may reflect the distinct physiology of the two ventricles. From an autonomic perspective, SCZ is consistently associated with cardiac autonomic dysregulation (73, 74), and the RV may be more sensitive than the LV to cardiopulmonary-autonomic coupling because it operates in the low-pressure pulmonary circulation and is more responsive to neurohumoral changes, venous return, and pulmonary vascular load (63, 75–78). From a hemodynamic perspective, RV structure may more closely reflect pulmonary circulation, oxygenation, and CO_2_-related cerebrovascular regulation (63, 77, 79–81), whereas LV traits are more strongly influenced by systemic arterial load and cardiometabolic factors (82, 83). Accordingly, ESV of RV may capture a SCZ-related physiological axis that is less apparent in LV phenotypes. This interpretation should be considered hypothesis-generating rather than definitive, and the inverse association observed here should not be taken to indicate that higher ESV of RV is clinically protective per se.

Alternatively, the observed cardiocerebral association may reflect a shared genetic architecture, in which common genetic variants influence both cardiac structure and SCZ risk (12). While the exact biological pathways remain unclear, it is plausible that some shared genetic influences affect both systems through complex regulatory mechanisms (12, 13). This perspective highlights a potential direction for future mechanistic studies without making specific claims about individual genes or pathways.

Nevertheless, these interpretations should be considered in the context of the relatively small MR effect estimates observed in the present study. In both the UKB dataset and the GWAS summary statistics, ESV of RV was originally reported on a 1 mL scale, which contributed to the modest effect sizes observed in the MR analysis. However, the physiological variation range of ESV of RV in clinical practice is substantially broader, typically ranging from approximately 13 to 91 mL in healthy individuals and potentially exceeding this range in patients with cardiovascular disease (84). Therefore, the MR-derived effect estimates may have limited direct clinical interpretability. Importantly, the statistical significance of the observed association should primarily be interpreted as evidence suggestive of a potential relationship between ESV of RV and SCZ, rather than as evidence supporting a clinically meaningful protective effect of increased ESV of RV itself.

Compared with the primary analysis, the statistical evidence for the reverse causal direction was relatively weak. The stronger forward direction is suggestive that genetic variants influencing cardiac structure may have more direct downstream consequences for neurodevelopment than the reverse. This asymmetry suggests that genetic influences on cardiac structure may represent the predominant direction contributing to this association (85, 86). If SCZ exerted a substantial reverse causal effect on the cardiac structure, we would generally expect to observe more robust MR evidence, assuming adequate instrument strength. However, the weak association currently observed is plausibly attributable to genetic correlation arising from pleiotropic genes, rather than to a strong direct causal effect of SCZ on cardiac structure (86). Overall, these findings are consistent with the forward MR results and support an association between genetically predicted ESV of RV and SCZ risk.

Based on the present analysis, this study provides evidence suggesting a potential “heart-to-brain” association between ventricular structure alterations and the risk of SCZ from the perspective of genetic causality. Among the findings, genetically predicted ESV of RV shows a significant inverse association with SCZ risk in forward MR analysis. This finding suggests a possibility that the physiological factors, including cardiac structure, may contribute to SCZ risk. Furthermore, the analyses provide a new direction for clarifying how cardiac structural variation may relate to brain health and also may provide a basis for future research exploring cardiopsychiatric interactions and potential cross-system mechanisms related to SCZ. Overall, this study broadens the perspective on the “heart-to-brain” relationship and offers new physiological and genetic considerations for etiological research on SCZ.

In the present study, the implementation of multiple complementary strategies to minimize potential bias due to horizontal pleiotropy, including confounder-associated SNP exclusion, functional annotation and pathway-based filtering, pleiotropy-robust MR methods, Steiger directionality filtering, and MVMR analyses adjusting for correlated psychiatric liabilities. Collectively, these approaches strengthened the validity of the MR assumptions, reduced the likelihood that the observed associations were driven by alternative biological pathways or residual confounding, and improved the overall credibility and interpretability of the causal estimates. However, several scientifically reasonable boundaries of our study warrant consideration. First, the present findings from the UKB and MR analyses may still be influenced by unknown confounding factors, and replication in independent datasets will be necessary to further validate our results. Second, our study population was predominantly composed of individuals of European ancestry from the UKB. The generalizability of the findings may therefore be limited, and further studies in populations with diverse genetic backgrounds and environmental exposures are warranted. Third, given the relatively small number of clinically diagnosed SCZ cases in the UKB, we used SCZ PRS as a surrogate for genetic susceptibility to SCZ. Although PRS is a widely validated tool for quantifying genetic liability, it may not fully capture the clinical heterogeneity or environmental influences associated with clinically diagnosed SCZ. Therefore, the UKB findings should be interpreted as associations with genetic liability to SCZ rather than direct associations with clinically confirmed disease. Fourth, although multiple sensitivity analyses and pleiotropy screening procedures were performed, residual horizontal pleiotropy cannot be completely excluded, which may still influence the MR estimates. Fifth, although multivariable adjustments were performed, the unequal distribution of sex and other baseline covariates may still have contributed to residual confounding and could potentially influence the generalizability of the findings. Finally, ventricular CMR traits were obtained at a single time point, which limits the assessment of dynamic changes in cardiac structure over time and their relationship to neuropsychiatric risk. This cross-sectional design reflects the limitations of the current data collection framework, highlighting the importance of longitudinal designs in future studies to capture time-dependent brain-heart interaction. Future studies can build on these boundaries by incorporating multi-ethnic cohorts to enhance generalizability, adopting longitudinal designs to track dynamic cardiac structural changes, and using longitudinal imaging or experimental models to further explore the connections between cardiac structure and neuropsychiatric disorders.

## Conclusion

In summary, UKB and MR analyses identified an inverse association between genetically predicted ESV of RV and the risk of SCZ. These findings support a potential relationship between ventricular cardiac structure and SCZ risk from a heart-to-brain perspective and expand the current understanding of heart-brain interactions in psychiatric disorders. The findings may contribute to future strategies for early risk identification and interdisciplinary prevention research in SCZ. Further longitudinal and mechanistic studies are warranted to validate these findings and clarify their biological and clinical relevance.

## Data Availability

Publicly available datasets were analyzed in this study. This research was conducted using the UK Biobank Resource under approved application number [194287]. The UK Biobank data are available to bona fide researchers upon application and approval by UK Biobank (https://www.ukbiobank.ac.uk/). Restrictions apply to the availability of these data, which were used under license for the current study and are therefore not publicly available. Summary-level data generated during this study are available from the corresponding author upon reasonable request. Publicly available GWAS summary statistics for SCZ and CMR traits were obtained from published studies and accessed through their respective repositories. GWAS summary statistics for ventricular CMR traits were obtained from Schmidt et al. (41). GWAS summary statistics for SCZ were obtained from Trubetskoy et al. (42).
