# Supplementary material for: Ventricular cardiac magnetic resonance traits and schizophrenia risk: a UK Biobank and Mendelian randomization study
Source: Front Psychiatry. 2026 Jun 12;17:1766780. doi: 10.3389/fpsyt.2026.1766780 (PMC13303614; doi:10.3389/fpsyt.2026.1766780)
Supplement: Supplementary file 1 [file Image1.pdf]

Figure S1. Leave-one-out sensitivity analysis for the causal association between ESV of RV and SCZ.

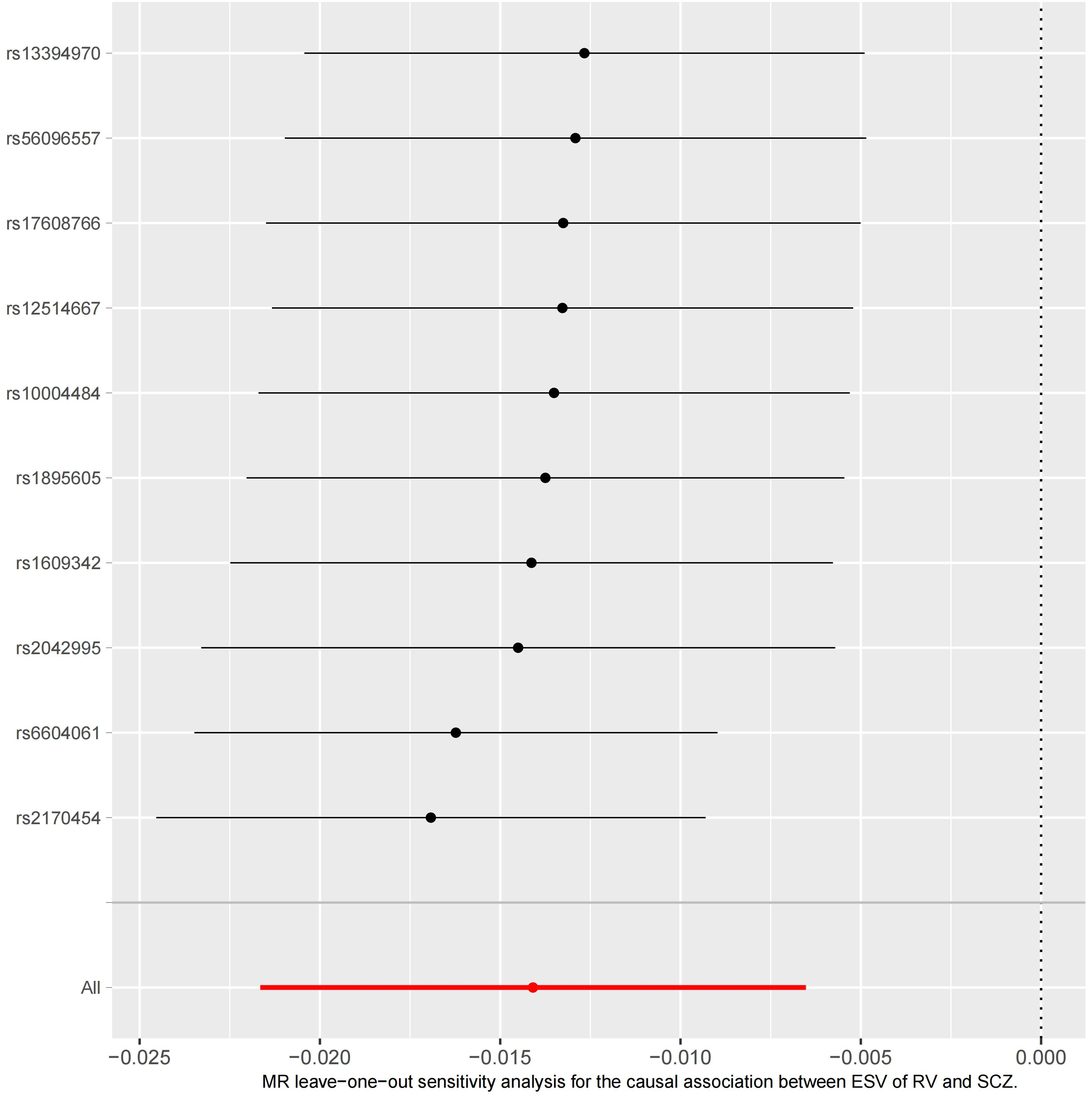

Abbreviations: ESV, end systolic volume; RV, right ventricle; SCZ, schizophrenia; MR, Mendelian randomization.

Figure S2. Forward MR analysis using IVs selected at P < 1 × 10^{-7}.

Exposure	Method	No. of SNP	OR(95% CI)	*P*
EDV of LV	IVW	3	1.01 (0.98 to 1.04)	0.699
	MR Egger	3	1.10 (0.94 to 1.27)	0.440
	Weighted median	3	1.00 (0.99 to 1.02)	0.662
	Weighted mode	3	1.00 (0.98 to 1.02)	0.913
EF of LV	IVW	11	1.01 (0.99 to 1.03)	0.157
	MR Egger	11	0.98 (0.90 to 1.06)	0.640
	Weighted median	11	1.01 (0.99 to 1.03)	0.450
	Weighted mode	11	1.00 (0.97 to 1.04)	0.897
ESV of LV	IVW	13	1.00 (0.99 to 1.01)	0.849
	MR Egger	13	1.01 (0.97 to 1.06)	0.652
	Weighted median	13	1.00 (0.99 to 1.01)	0.663
	Weighted mode	13	1.00 (0.98 to 1.01)	0.939
EDV of RV	IVW	5	0.99 (0.99 to 1.00)	0.103
	MR Egger	5	1.01 (0.97 to 1.05)	0.715
	Weighted median	5	0.99 (0.98 to 1.00)	0.060
	Weighted mode	5	0.99 (0.98 to 1.00)	0.121
EF of RV	IVW	9	0.99 (0.98 to 1.02)	0.619
	MR Egger	9	1.04 (0.84 to 1.29)	0.712
	Weighted median	9	0.99 (0.96 to 1.02)	0.451
	Weighted mode	9	0.98 (0.94 to 1.03)	0.444
ESV of RV	IVW	11	0.99 (0.98 to 1.00)	0.002
	MR Egger	11	0.99 (0.95 to 1.03)	0.512
	Weighted median	11	0.99 (0.98 to 1.00)	0.008
	Weighted mode	11	0.98 (0.96 to 0.99)	0.024
SV of RV	IVW	2	1.01 (0.99 to 1.03)	0.562

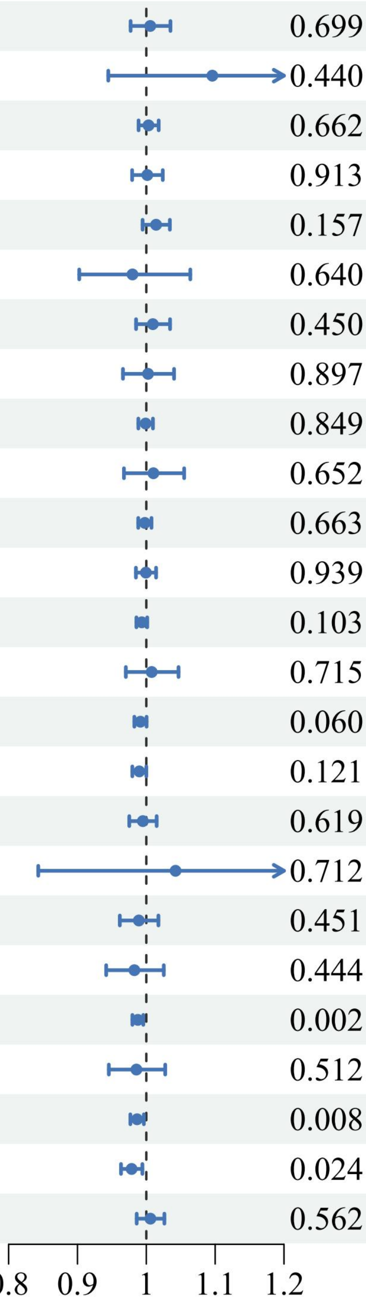

Abbreviations: IV, instrumental variable; SNP, single nucleotide polymorphism; OR, odds ratio; CI, confidence interval; EDV, end diastolic volume; EF, ejection fraction; ESV, end systolic volume; SV, stroke volume; LV, left ventricle; RV, right ventricle; IVW, inverse-variance weighted; MR, Mendelian randomization.

Figure S3. Forward MR analysis using IVs selected at P < 1 × 10-6.

Exposure	Method	No. of SNP	OR(95% CI)	*P*
EDV of LV	IVW	13	1.00 (1.00 to 1.01)	0.342
	MR Egger	13	1.01 (0.97 to 1.05)	0.721
	Weighted median	13	1.00 (1.00 to 1.01)	0.255
	Weighted mode	13	1.01 (0.99 to 1.02)	0.466
EF of LV	IVW	18	1.02 (1.00 to 1.04)	0.124
	MR Egger	18	0.97 (0.90 to 1.06)	0.514
	Weighted median	18	1.01 (0.99 to 1.03)	0.515
	Weighted mode	18	1.00 (0.97 to 1.03)	0.896
ESV of LV	IVW	22	1.00 (0.99 to 1.01)	0.577
	MR Egger	22	0.99 (0.96 to 1.03)	0.734
	Weighted median	22	1.00 (0.99 to 1.01)	0.425
	Weighted mode	22	1.00 (0.98 to 1.01)	0.726
SV of RV	IVW	4	0.99 (0.97 to 1.00)	0.141
	MR Egger	4	1.01 (0.97 to 1.06)	0.635
	Weighted median	4	0.99 (0.98 to 1.01)	0.273
	Weighted mode	4	1.00 (0.97 to 1.03)	0.980
EDV of RV	IVW	15	1.00 (0.99 to 1.00)	0.552
	MR Egger	15	0.99 (0.97 to 1.01)	0.293
	Weighted median	15	1.00 (0.99 to 1.00)	0.391
	Weighted mode	15	1.00 (0.98 to 1.01)	0.591
EF of RV	IVW	14	1.00 (0.98 to 1.02)	0.900
	MR Egger	14	0.97 (0.89 to 1.06)	0.566
	Weighted median	14	1.00 (0.98 to 1.03)	0.823
	Weighted mode	14	0.99 (0.95 to 1.03)	0.739
ESV of RV	IVW	17	0.99 (0.98 to 1.00)	0.105
	MR Egger	17	0.98 (0.95 to 1.02)	0.399
	Weighted median	17	0.99 (0.98 to 1.00)	0.013
	Weighted mode	17	0.98 (0.97 to 1.00)	0.039
SV of RV	IVW	4	1.00 (0.99 to 1.02)	0.585
	MR Egger	4	1.01 (0.94 to 1.08)	0.867
	Weighted median	4	1.01 (0.99 to 1.02)	0.524
	Weighted mode	4	1.01 (0.99 to 1.04)	0.402

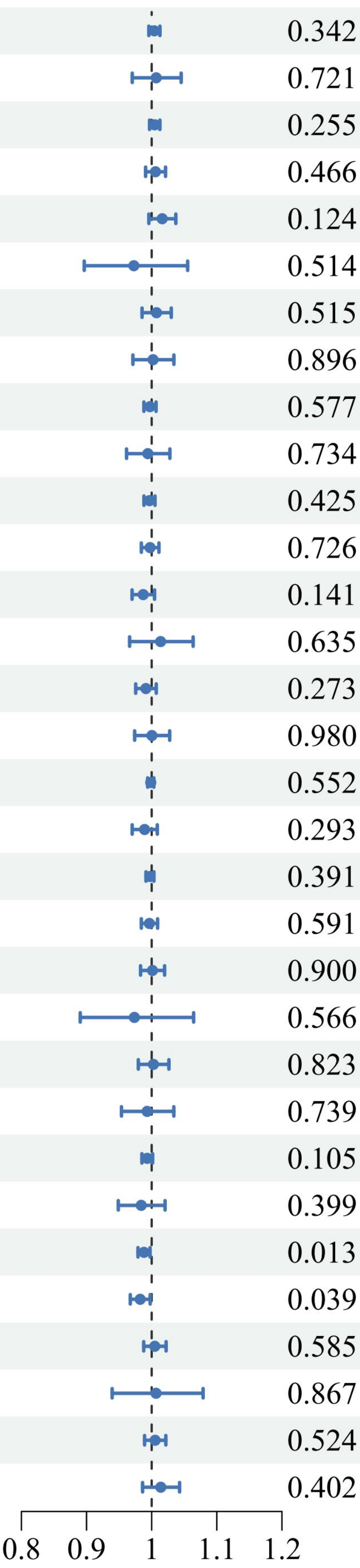

Abbreviations: IV, instrumental variable; SNP, single nucleotide polymorphism; OR, odds ratio; CI, confidence interval; EDV, end diastolic volume; EF, ejection fraction; ESV, end systolic volume; SV, stroke volume; LV, left ventricle; RV, right ventricle; IVW, inverse-variance weighted; MR, Mendelian randomization.
